# Phylogeography and epidemic history of hepatitis C virus genotype 4 in Africa

**DOI:** 10.1016/j.virol.2014.07.006

**Published:** 2014-09

**Authors:** James C. Iles, Jayna Raghwani, G.L. Abby Harrison, Jacques Pepin, Cyrille F. Djoko, Ubald Tamoufe, Matthew LeBreton, Bradley S. Schneider, Joseph N. Fair, Felix M. Tshala, Patrick K. Kayembe, Jean Jacques Muyembe, Samuel Edidi-Basepeo, Nathan D. Wolfe, Peter Simmonds, Paul Klenerman, Oliver G. Pybus

**Affiliations:** a Department of Zoology, University of Oxford, South Parks Road, Oxford, OX1 3PS, UK; b Department of Infection & Immunity, Walter & Eliza Hall Institute, Victoria 3052, Australia; c Department of Microbiology and Infectious Diseases, Université de Sherbrooke, Sherbrooke, Canada; d Global Viral, Yaoundé, Cameroon; e Metabiota, San Francisco, USA; f Department of Military Health, Ministry of Defense, Kinshasa, Democratic Republic of the Congo; g Kinshasa School of Public Health, Kinshasa, Democratic Republic of the Congo; h National Institute of Biomedical Research, Kinshasa, Democratic Republic of the Congo; i National AIDS Control Program, Reference Laboratory, Kinshasa, Democratic Republic of the Congo; j Stanford University Program in Human Biology, Stanford, USA; k Roslin Institute, University of Edinburgh, Edinburgh, UK; l Peter Medawar Building for Pathogen Research, University of Oxford, Oxford, UK

**Keywords:** Hepatitis C, Molecular epidemiology, Prevalence, Phylogeny, Molecular clock, Skyline plot, Africa, DRC, Egypt

## Abstract

HCV genotype 4 is prevalent in many African countries, yet little is known about the genotype׳s epidemic history on the continent. We present a comprehensive study of the molecular epidemiology of genotype 4. To address the deficit of data from the Democratic Republic of the Congo (DRC) we PCR amplified 60 new HCV isolates from the DRC, resulting in 33 core- and 48 NS5B-region sequences. Our data, together with genotype 4 database sequences, were analysed using Bayesian phylogenetic approaches. We find three well-supported intra-genotypic lineages and estimate that the genotype 4 common ancestor existed around 1733 (1650–1805). We show that genotype 4 originated in central Africa and that multiple lineages have been exported to north Africa since ~1850, including subtype 4a which dominates the epidemic in Egypt. We speculate on the causes of the historical intra-continental spread of genotype 4, including population movements during World War 2.

## Highlights

• A comprehensive study of the molecular epidemiology of HCV genotype 4.
• PCR amplification of 60 HCV+ isolates from the DRC.
• We estimate the genotype 4 common ancestor existed between 1650 and 1805.
• We show that genotype 4 originated in central Africa.
• Multiple lineages were exported to north Africa since ~1850, including subtype 4a.

## Introduction

Hepatitis C virus (HCV) is a major human pathogen that causes substantial morbidity and mortality worldwide. It is estimated that more than 185 million people are chronically infected with HCV and that there are 3–4 million new infections each year (Mohd Hanafiah et al., 2013). Infection with the virus is typically asymptomatic or unspecific in the initial stages, but once it progresses to long-term chronic infection it can lead to liver cirrhosis, fibrosis, and sometimes hepatocellular carcinoma (Lauer and Walker, 2001).

HCV is a genetically diverse virus that is classified into seven genotypes (1–7) with an average of 35% nucleotide divergence between strains belonging to different genotypes. All genotypes except 5 and 7 are subdivided into numerous subtypes (1a, 1b, 1c, 2a, 2b etc.) and the average nucleotide divergence between subtypes of the same genotype is around 25% (Murphy et al., 2007a, Simmonds et al., 1993, Smith et al., 2014).

Although the majority of HCV infections worldwide are caused by a small number of ‘epidemic’ subtypes (e.g. 1a, 1b, 2a and 3a) that spread rapidly during the twentieth century (Magiorkinis et al., 2009, Pybus et al., 2005, Simmonds, 2004), there are clear geographic patterns in the distribution of HCV genetic diversity. Highly divergent ‘endemic’ strains that belong to the same genotype are typically found in a restricted geographic area, indicating the presence of the genotype in that location for hundreds or thousands of years (Simmonds, 2004). For example, HCV genotype 2 is thought to be endemic in West Africa, genotypes 1 and 4 in Central Africa and the Middle East and genotype 6 in East Asia (Candotti et al., 2003, Jeannel et al., 1998, Mellor et al., 1995, Ndjomou et al., 2003, Pybus et al., 2009).

The epidemiology of HCV prior to the discovery of the virus is poorly understood. Documentary evidence of past HCV transmission is difficult to establish as symptoms during acute infection are unspecific and HCV incidence before the widespread use of injections was likely too low to create notable outbreaks of disease. Further, samples available for retrospective screening that were archived before the 1970s are exceptionally rare (Gray et al., 2013). As a consequence, evolutionary analyses of contemporary HCV gene sequences using phylogenetic and coalescent-based methods have been utilised to estimate dates of viral divergence and to estimate viral effective population size through time. In addition, previous studies of genotype 2 in Africa (Markov et al., 2009) and of genotype 6 in Asia (Pybus et al., 2009) employed phylogeographic and molecular clock methods and provided insights into the historical geographic spread of HCV, the age of HCV genotypes and subtypes, and their recent transmission history.

To date there has been no systematic phylogeographic or evolutionary study of HCV genotype 4 as a whole. This genotype is common throughout most of Central Africa and parts of the Middle East. Recent estimates indicate that there are ~8 million people infected with HCV in Central and Eastern Sub-Saharan African, and >15 million people infected across North Africa and the Middle East (Mohd Hanafiah et al., 2013). Genotype 4 (and subtype 4a in particular) dominates the HCV epidemic in Egypt, where 15% of adults are antibody-positive for HCV, with a much higher prevalence seen in older cohorts (El-Zanaty and Way, 2009). HCV in Egypt has been described as a ‘local epidemic’, whereby the transmission of one or a few subtypes rises rapidly within a region, but without the international dissemination observed for the ‘global epidemic’ subtypes such as 1a and 1b. High HCV seroprevalences and local epidemics associated with other subtypes of genotype 4 and have also been reported in many sub-Saharan African countries. For example, 11.2% of people screened in rural Gabon were seropositive for HCV (Njouom et al., 2012) of whom 92% were infected with genotype 4. In that study major risk factors for HCV infection were past injections, hospital admissions, and age greater than 55. In Cameroon, HCV seroprevalence was 11% in a group of high-HIV risk individuals and 16% of the HCV infections were classified as genotype 4 (Ndjomou et al., 2003). In a separate Cameroonian cohort comprising individuals aged over 60, HCV seroprevalence was 56% and 54% of infections were genotype 4 (Pépin et al., 2010a). In each of these studies HCV seroprevalence was strongly associated with age and subtypes 4a and 4r were observed.

The evolution and genetic history of genotype 4 is worthy of investigation for several reasons. First, together with genotype 1, genotype 4 responds less well to interferon-based anti-HCV drug treatment than genotypes 2 and 3, especially in patients of African descent (Chen et al., 2012, Rose et al., 2013) and it has been hypothesised that this phenotype is a consequence of the long-term presence of genotypes 1 and 4 in Central African populations (Rose et al., 2013). Second, there are a number of unanswered questions concerning the origin and spread of HCV genotype 4 within Africa. For example, the current distribution and past spread of genotype 4 strains among countries is unclear and the geographic source of the HCV lineages present in Egypt and the Middle East is currently unknown. In addition, in recent years there has been rapid growth in the prevalence of HCV subtypes 4a and 4d in Europe, particularly among injecting drug users (IDUs), hence a comprehensive overview of genotype 4 diversity may prove useful for public health assessments outside Africa (Ciccozzi et al., 2012, De Bruijne et al., 2009, van Asten et al., 2004).

The investigation of HCV evolution in Central Africa is hampered by a lack of information about its epidemiology and genetic diversity in the region. Mohd Hanafiah et al. (2013) define the evidentiary support for HCV prevalence in the region as ‘very limited’. Although recent surveillance studies have explored the genetic diversity of HCV in Cameroon, Gabon and the Republic of Congo (Cantaloube et al., 2010, Ndong-Atome et al., 2008, Nerrienet et al., 2005, Njouom et al., 2007, Njouom et al., 2012) there is little information about the diversity of the virus in the Democratic Republic of the Congo (DRC). This country is the second largest in Africa and has 67 million residents, making it the third most populous in the continent. However, the ongoing conflict there since 1996 has made disease surveillance difficult. The large size and central position of the DRC within Central Africa mean that phylogeographic studies of HCV in the region will be incomplete without a comprehensive survey of viral diversity in the country. Further, it is possible that the DRC harbours previously-undetected variants of the virus: the only published isolate of HCV genotype 7 was isolated from a Canadian resident who had emigrated from the DRC (Murphy et al., 2007b).

At present there is little information about the genetic diversity of HCV infections in the DRC. In a previous small-scale survey of blood samples from the country (Iles et al., 2013) we detected HCV RNA in 11 individuals. Phylogenetic analysis of HCV core and NS5B region sequences from these samples indicated that they belonged to several classified and unclassified subtypes of genotype 4. In this study we address the deficit of HCV genetic information from the DRC with the screening and sequencing of 1999 blood samples from the country. We combine these new data with genotype 4 sequences gathered from online databases and originating from countries across Africa and worldwide. This enables us to analyse the DRC samples in context with the larger diversity of HCV genotype 4 viruses and to investigate the long-term evolutionary history of the virus within the African continent.

## Results

### Age distribution

Of the 1999 samples tested, 3% (*n*=60) were positive for HCV 5′UTR RNA. Of these 60 samples, 33 produced core sequence and 48 produced NS5B sequence. These results are broadly consistent with our pilot study which detected HCV RNA in 3.7% (*n*=11) of samples and yielded 9 core sequences and 11 NS5B sequences (Iles et al., 2013).

Fig. 2 shows the age distribution of HCV RNA positivity in our population, (including data from the pilot study). Samples were scored as HCV RNA positive if sequence was obtained from at least one of the 5′UTR, core or NS5B regions. When grouped according to date of birth, samples from older individuals are more likely to contain HCV RNA than younger ones; the highest prevalence (9.2%) was observed in the 1930–1945 cohort. Samples from patients born in or before 1945 were significantly more likely to contain HCV RNA than those born after (*p*<0.001 using Fisher׳s exact test).

**Fig. 1:**
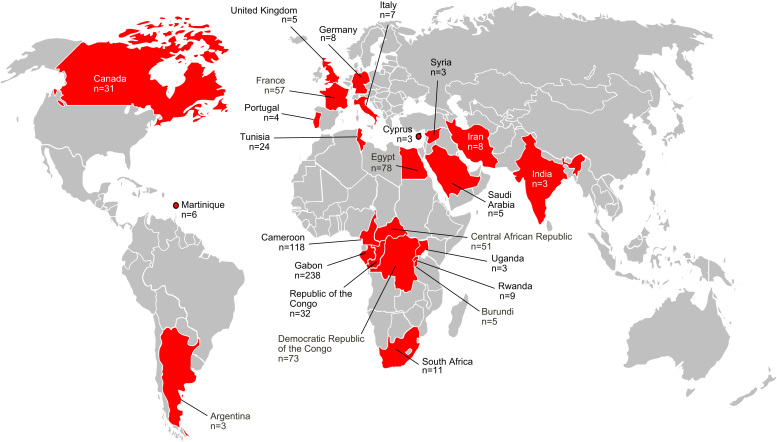
Map of the world showing the country of origin and sample size of samples used in this study. Where a single isolate yielded multiple sequences, they are only counted as one sample on this figure. Countries less than three samples are not shown.

**Fig. 2:**
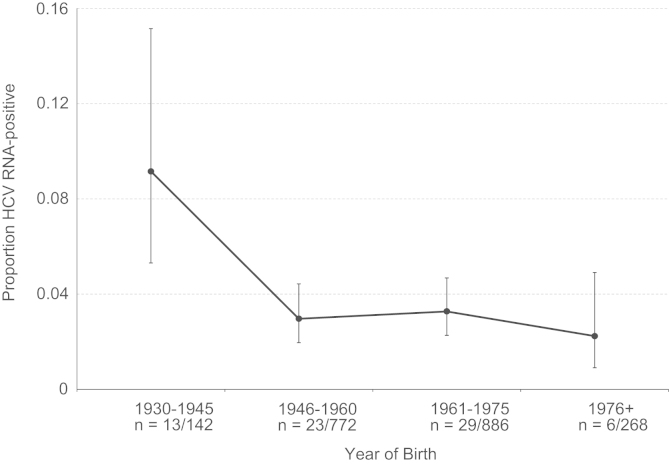
The age distribution of HCV RNA positivity among 2298 blood samples from the DRC. Samples are assigned to one of four age categories by year of birth. Numbers below each category indicate the number of positive samples/total number of samples. Fifteen samples (all negative) did not have date of birth information and are not included. The *y*-axis shows the proportion of samples in each age category that were HCV RNA-positive. The errors bars represent 95% conﬁdence limits of this proportion, estimated using the adjusted Wald method (Agresti and Coull, 1998).

### Phylogenetic analysis

Maximum-likelihood phylogenies estimated from the core and NS5B alignments are too large to display here and are thus provided in Supplementary Figs. 1 and 2. Sequences from all formally defined subtypes of genotype 4 (Smith et al., 2014) were present in the alignments, as well as sequences from subtypes that are not formally defined due to a lack of whole-genome reference sequences (e.g. 4e, 4h, and 4u). The numbers of sequences assigned to each subtype and the most common locations of sampling of each subtype are shown in Table 3. A total of 74 samples were genetically too divergent to be assigned to a known subtype, 26 of which appear to belong to a provisional subtype circulating in the Central African Republic (referred to here as 4car). Sequences from five isolates (including one obtained in this study) were discordant, i.e. they grouped into different subtypes in the core and NS5B alignments. The HCV-positive samples sequenced in this study were genetically diverse and were classified as belonging to subtypes 4c (*n*=17), 4h (*n*=2), 4k (*n*=18), and 4r (*n*=8). One sample (DRC0387) could not be classified into any known subtype but grouped with two unclassified isolates (DRC2431 and DRC2450) from our pilot study (Iles et al., 2013). This cluster of three strains is denoted 4drc here and represents a potentially new subtype (see Table 3). One sample (isolate DRC1427) generated a core sequence that was classified as 4q and a NS5B sequence classified as 4c. Few nodes in the core and NS5B maximum likelihood trees had high bootstrap support (Supplementary Figs. 1 and 2), but that is not unexpected for phylogenies estimated from these short subgenomic regions (as previously noted in Pybus et al., 2009 and elsewhere).

We discerned four clusters (denoted C1, C2, C3 and C4) that contained multiple sequences from our study population (Supplementary Figs. 1 and 2). Cluster C1 was present in subtype 4c in both the core and NS5B trees and contained 10 new DRC isolates (plus one from the pilot study). Cluster C2 was also found in subtype 4c and comprised six isolates from this study in the NS5B tree (only four of which are present in the core tree). Cluster C3 contained 12 subtype 4k samples from this study (plus two from the pilot study). C3 also included 15 samples from Tunisia in the NS5B tree, whereas in the core tree it included seven sequences from Gabon. Finally, cluster C4 was present in subtype 4r. In the NS5B tree C4 contained eight sequences from this study (plus two from the pilot study).

Samples from our study population also appeared outside of these four clusters. Specifically, two further isolates were placed inside subtype 4c, nine within 4k, two within 4h, one within 4q and three grouped in the unclassified lineage 4drc (see above). The pair of subtype 4h sequences grouped significantly, together with a strain from Brazzaville (Republic of the Congo; GU088141).

### Whole genome phylogenies

**Fig. 3:**
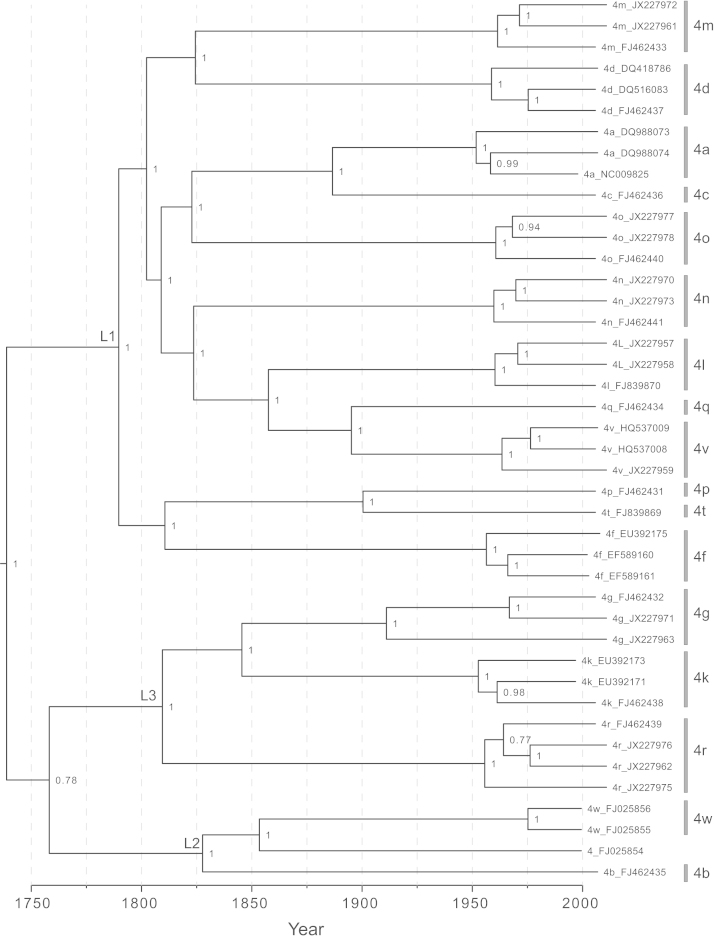
Maximum clade credibility molecular clock phylogeny, estimated from the whole genome alignment. Branch lengths represent time (see scale bar at the bottom of the figure). Posterior clade probabilities are shown next to each node. Sequences are labelled with their subtype and accession number. Subtypes are indicated on the right side of the diagram. The three intra-genotypic lineages discussed in the main text are labelled L1, L2, and L3.

Fig. 3 shows a maximum clade credibility (MCC) phylogeny of HCV genotype 4, obtained from the Bayesian molecular clock analysis of the whole genome sequences. An equivalent maximum likelihood phylogeny is provided in Supplementary Fig. 3. Up to three genomes per subtype were included and no complete genome sequences were available for some subtypes (subtypes 4e, 4h, 4s and 4u). As Fig. 3 indicates, there is a great deal of phylogenetic structure above the subtype level. This structure is supported by high posterior probability values in the Bayesian phylogeny and by high bootstrap values in the ML phylogeny (Fig. 3; Supplementary Fig. 3).

Three intra-genotypic lineages can be discerned and are denoted here L1, L2, and L3. These lineages correspond to the three clades closest to the root of genotype 4 with strong statistical support. Lineage L1 contains subtypes 4a, 4c, 4d, 4l, 4m, 4n, 4o, 4q and 4v, L2 contains subtypes 4b and 4w and L3 contains subtypes 4g, 4k, and 4r. In the ML phylogeny, L2 is present as an outgroup (Supplementary Fig. 3) whereas in the Bayesian tree it is placed as a sister group to L3, albeit with a comparatively low posterior probability of 0.78 (Fig. 3).

The Bayesian molecular clock analysis provided an estimate of the date of the most recent common ancestor (MRCA) of genotype 4, which was 1733 (95% HPD credible region=1650–1805). This date is more recent than some previous estimates for the origin of genotype 4. Pybus et al. (2001) estimated HCV evolutionary rates from a small data set of dated sequences and used these to infer that the MRCA of genotype 4 existed about 350 years ago. Njouom et al. (2007) used the same rate estimates during a more comprehensive Bayesian phylogenetic analysis and dated the MRCA of genotype 4 to 1541 (95% CIs: 1343–1698). The more recent date estimated here is likely to be more accurate because (i) it is based on whole genome sequences rather than small subgenomic fragments and (ii) it employs new HCV evolutionary rates that were estimated using larger data sets of dated sequences and more powerful methods of analysis (Gray et al., 2011). We note that the MRCA date provided here could be underestimated as a result of strong purifying selection (Wertheim and Kosakovsky Pond, 2011) or overestimated due to strong among-branch rate variation (Wertheim et al., 2012). However, Markov et al. (2012) showed that molecular clock estimates of HCV lineage movement between Africa and the Americas matched the known timeframe of the trans-Atlantic slave trade. This suggests that among-subtype HCV divergence dates within a genotype can be estimated with reasonable accuracy.

### Concatenated alignment phylogenies

**Fig. 4:**
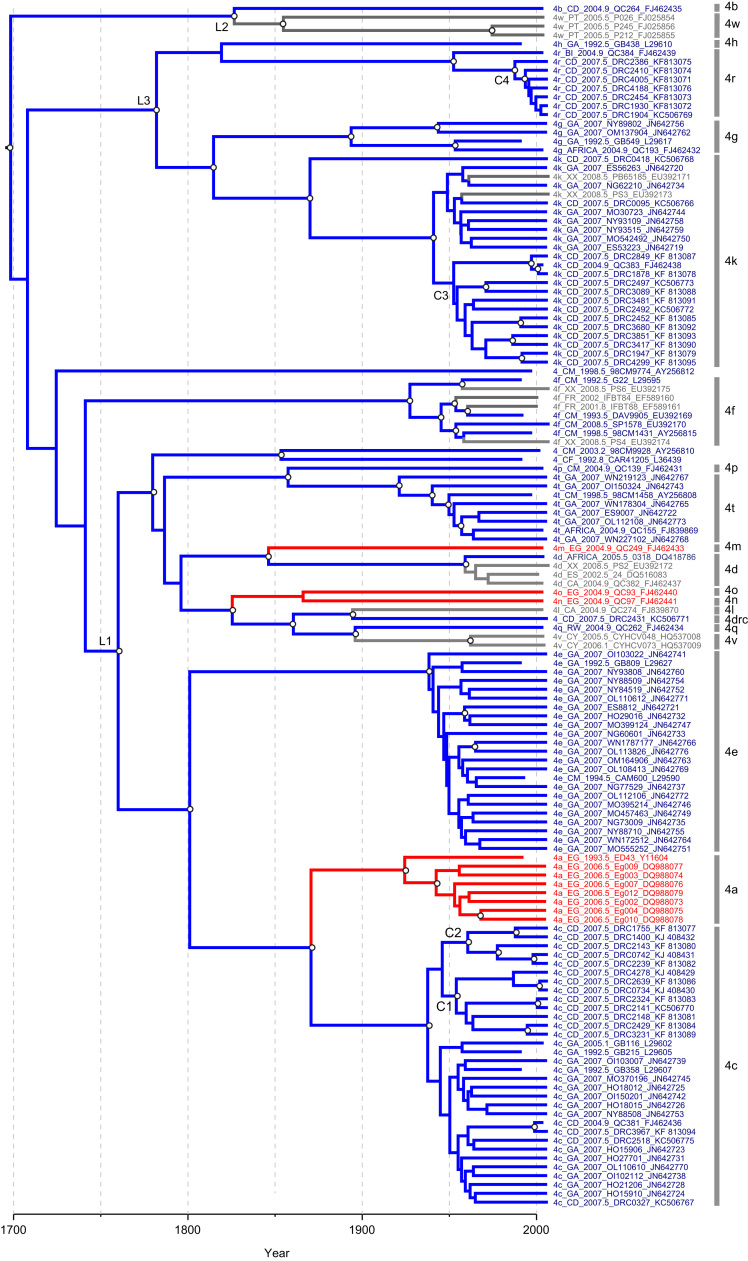
Maximum clade credibility molecular clock phylogeny, estimated from the concatenated alignment. Taxa are coloured according to location of sequence origin (blue=sub-Saharan Africa; red=Middle East and North Africa; grey=rest of the world). The locations of internal branches were inferred using parsimony and are coloured similarly. Branch lengths represent time (see scale bar at the bottom of the figure). Nodes with a posterior probability >0.9 are labelled with a white circle. Sequences are labelled as follows: subtype, sampling location using two-letter country codes (ISO 3166; see Table 4), sampling date, isolate name, accession number. XX represents an unknown location. Subtypes are indicated on the right side of the diagram. The three intra-genotypic lineages discussed in the main text are labelled L1, L2, and L3. The four clusters of samples obtained in this study discussed in the main text are labelled C1, C2, C3, and C4.

Fig. 4 shows the MCC phylogeny obtained from the Bayesian molecular clock analysis of the concatenated sequences (core plus NS5B). As in the whole genome phylogenies, all subtypes were monophyletic, and three well-supported intra-genotypic lineages were again observed. The greater number of subtypes present in concatenated analysis indicates that L1 contains subtypes 4a, 4c, 4d, 4e, 4l, 4m, 4n, 4o, 4p, 4q, 4t and 4v, L2 contains subtypes 4b and 4w, and lineage L3 contains subtypes 4g, 4h, 4k and 4r. L2 is again placed in a basal position (as in the full-genome ML phylogeny; Supplementary Fig. 3). However the weak posterior probability for this placement (0.21) suggests that L2 may be in reality be a sister group to L3 (as in the full genome Bayesian phylogeny; Fig. 3). Subtype 4f plus one divergent isolate from Cameroon (98CM9774) did not group into any of these three well supported intra-genotypic lineages.

The concatenated alignment yielded an estimate of the date of the genotype 4 common ancestor of 1687 (95% HPD credible region: 1538, 1811). This estimate overlaps considerably with the estimate from the whole genome alignment and the upper 95% HPD limits of both are almost identical. The credible region for the concatenated alignment is wider than that for the whole genome alignment, likely reflecting the reduced phylogenetic information in the former.

The molecular clock analysis was also used to estimate the date of origin of the four sequence clusters (C1–4) that contained isolates from our study population (see above). The estimated date of the MRCA of cluster C1 was 1953 (95% HPD: 1938, 1982). For cluster C2 the MRCA was dated to 1960 (95% HPD: 1930, 1976) and for cluster C3 it was 1952 (95% HPD: 1928, 1975). The estimated MRCA for cluster C4 was somewhat more recent and was dated to 1987 (1976, 2000). While these clusters had low bootstrap scores in the ML trees (Appendix A, Appendix A), in this analysis C1, C2 and C4 are all supported with posterior probabilities >0.9, while C3 has a posterior probability of 0.79. This increased statistical support is likely due to the combined phylogenetic signal in the concatenated analysis as compared to that available in the ML reconstruction of individual genome regions.

The taxa and branches of Fig. 4 have been coloured according to the known or inferred country of origin for each isolate. The majority of strains are from sub-Saharan Africa (blue). Sequences from North Africa/Middle East (red) and from the rest of the world (grey) cluster together *within* the greater diversity of genotype 4 from sub-Saharan Africa. Thus it is clear that genotype 4 originated in central Africa before disseminating elsewhere. Further, some isolates without location information or which were sampled outside Africa (e.g. subtypes 4f and 4k strains closely related to those from Cameroon and Gabon) may also represent infections that were acquired in Central Africa. Almost all isolates sampled *outside* central Africa belong to the intra-genotypic lineage L1 (Fig. 4). Sequences from Egypt are commonly found in subtypes 4a, 4l, 4m, 4n and 4u (Appendix A, Appendix A; Table 3). Subtype 4d is currently found in many countries, especially in Western Europe, where it is prevalent among some injecting drug user populations (De Bruijne et al., 2009).

Fig. 4 provides some clues as to the origin of the most common subtypes of genotype 4. For example, subtype 4a is found worldwide but is highly prevalent in Egypt, where it was likely spread by widespread injection treatment campaigns during the mid-twentieth century (e.g. Strickland, 2006, Pybus et al., 2003). Using our results we can explore the question of from where the Egyptian HCV epidemic originated. Our analysis indicates that subtypes 4a and 4c diverged from each other around 1870 and that subtype 4c mainly comprises sequences from Gabon and the DRC. In the core and NS5B trees (Appendix A, Appendix A) we can observe strains sampled from Cameroon and the Central African Republic that are phylogenetically immediately basal to subtypes 4a, 4n and 4o. Fig. 4 indicates that these subtypes (and subtype 4m) arrived in Egypt from Central Africa no earlier than 1825.

Fifteen subtype 4k isolates from Tunisia cluster together within the much greater diversity of subtype 4k sampled from central Africa. The Tunisian strains are closely related to DRC isolates from this study belonging to cluster C3. Although these Tunisian samples were not included in the concatenated analysis (because only NS5B sequences were available) we can combine Appendix A, Appendix A to conclude that the Tunisian subtype 4k infections originated from central Africa (possibly from the DRC) at around the time of the MRCA of cluster C3, which was 1952 (1928, 1975). A second cluster of six unclassified Tunisian isolates can be observed in the NS5B phylogeny and form a sister lineage to subtype 4o (Supplementary Fig. 2).

### Bayesian skyline analysis

**Fig. 5:**
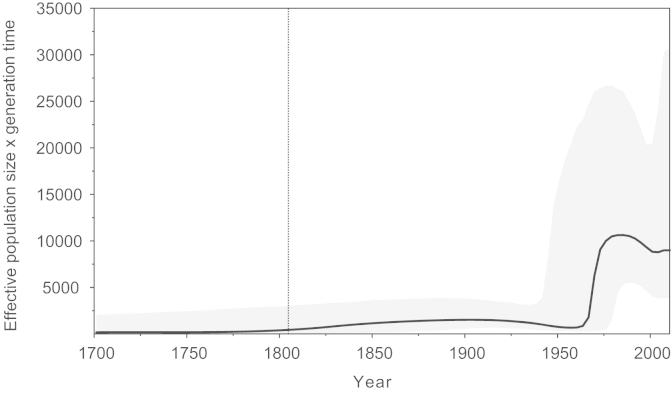
Bayesian skyline plot, showing the epidemic history of genotype 4 estimated from the concatenated alignment. The black line represents the estimated effective number of infections through time. The blue lines represent the 95% highest posterior density confidence intervals of this estimate. The earliest date in the plot is the median estimate of the TMRCA of genotype 4, while the dotted line shows the upper 95% highest posterior density confidence interval of this date.

The epidemic history of genotype 4 was investigated by estimating a Bayesian skyline plot from the concatenated alignment (Fig. 5). This plot represents the effective number of HCV infections through time, back to the estimated TMRCA of the genotype. Fig. 5 shows that prior to the twentieth century there was an extended period of endemic transmission of genotype 4, during which the effective number of infections was low. There is a sharp transition from endemic infection to rapid exponential growth starting around the 1950s. The epidemic history of genotype 4 after 1975 is much harder to discern due to very large confidence limits: either scenario after 1975 of continued growth or a stabilisation of prevalence are statistically compatible with the data. Previous studies of genotype 4 in several African countries (Central African Republic, Republic of Congo, Gabon, Cameroon) have noted similar, but earlier, transitions to rapid expansion, between 1930 and 1960 (Cantaloube et al., 2010, Njouom et al., 2007, Njouom et al., 2009, Njouom et al., 2012). The slightly later date of the epidemic transition reported here is likely explained by the new evolutionary rates used in our analysis, which are faster than those employed previously (see above for discussion). The overall shape of the epidemic curve presented in Fig. 5 closely resembles that previously estimated for genotype 6 in Asia (Pybus et al., 2009). However, as discussed in that paper, skyline plots that span entire HCV genotypes should be interpreted cautiously, as they exhibit geographic structure and combine subtypes that have experienced different rates of growth during the twentieth century (see Pybus et al., 2009 for further details). Thus only the broad qualitative trend of a twentieth-century transition to epidemic growth is likely to be robust.

## Discussion

In this study we report the first large-scale survey of HCV genetic diversity in the DRC, one of the geographically largest and most populous African countries. The previously unsurveyed state of the DRC represented a significant gap in our understanding of HCV in Africa and our results complement recent surveys of HCV in neighbouring countries, including Gabon, Cameroon, Republic of Congo and Central African Republic (e.g. Cantaloube et al., 2010, Njouom et al., 2007, Njouom et al., 2009, Njouom et al., 2012, Pépin et al., 2010a, Pépin et al., 2010b). We observed a high level of HCV genotype 4 diversity in our study population; isolates were classified as belonging to subtypes 4c, 4k, 4h and 4r, as well as to a potential new subtype circulating in the DRC (denoted 4drc). One isolate (DRC1427) was classified discordantly (4c/4q) in two sub-genomic regions sequenced and may represent a previously undetected recombinant strain. This diversity, together with the absence of globally-prevalent subtypes (e.g. 1a, 1b, and 3a), suggests that genotype 4 has been present in the DRC for a long period of time. However, the genetic diversity of HCV in the DRC is not as high as that in other central African countries where various subtypes of multiple HCV genotypes are sometimes observed (e.g. genotypes 1, 2 and 4 in Cameroon; Ndjomou et al., 2003, Pépin et al., 2010a). Further, we failed to detect any genotype 7 isolates despite using the same primers as those used to initially discover the strain (Murphy et al., 2007b), indicating that the prevalence of genotype 7 within the DRC is low. We conclude that the overall pattern of genetic diversity of HCV in the DRC is fundamentally different to that of HIV-1; the diversity of HIV-1 in the DRC is as large as that observed worldwide, implying that the virus originated in central Africa and its diversity elsewhere is reduced due to founder effects (Vidal et al., 2000, Rambaut et al., 2001), whereas we observed only one HCV genotype in the DRC.

Approximately 3% of our samples screened contained detectable HCV RNA, slightly less than the 5.2% and 6.4% found in HCV screening surveys in Gabon and Central African Republic respectively (Njouom et al., 2009, Njouom et al., 2012). Past studies of HCV in the DRC have only assayed seroprevalence, and have found anti-HCV in 6.4% of blood donors (Tibbs et al., 1991), 6.6% of sex workers and 4.3% of pregnant women (Laurent et al., 2001). These seroprevalence estimates are again somewhat less than those found in the Central African Republic (10.5%) and Gabon (11.2%) (Njouom et al., 2009, Njouom et al., 2012). Despite the large size of our survey, sampling was limited to males and therefore our prevalence estimates may not equal those of the general population. Cantaloube et al. (2010) found that men were 43% more likely to be seropositive than women in the Republic of the Congo, and it is possible that a similar pattern is true in the DRC. We confirmed that HCV positivity is significantly associated with older age in our study population (Fig. 2; Iles et al., 2013). Although the long-term persistence of most HCV infections means that older subjects are in general more likely to be infected, the notable increase in HCV prevalence in those born before 1945 suggests a distinct historical event. This ‘cohort effect’ has also been reported for HCV genotype 4 in Gabon, Cameroon and Egypt and is thought to be due to past iatrogenic transmission resulting from public health campaigns during the twentieth century that involved injections (Frank et al., 2000, Nerrienet et al., 2005, Njouom et al., 2007, Njouom et al., 2012). In the absence of any epidemiological data we cannot speculate further on the possible causes in our study population of the age effect. However, our reconstruction of the epidemic history of genotype 4 (Fig. 5) clearly shows a transition from low to high prevalence, likely representing the combined effects of iatrogenic transmission events in many different African countries during the middle of the twentieth century (Pépin and Labbé, 2008).

The molecular clock and phylogeographic results reported here clearly indicate that genotype 4 epidemics in North Africa, the Middle East and Europe all originated from central Africa (Fig. 4). Fig. 4 can be also used to estimate the time of exportation of lineages from central Africa to elsewhere; however, the ages of these movement events will often be overestimated due to the exclusion from the concatenated alignment of many central African strains that were sequenced in only the core or NS5B regions (Fig. 1, Fig. 2). Genotype 4 lineages present in Egypt are of particular interest, as the country has the highest prevalence of HCV worldwide (Frank et al., 2000, El-Zanaty and Way, 2009). We estimate that subtype 4a, which accounts for the majority of the Egyptian epidemic, arrived there from central Africa sometime between 1860 and 1925 (the estimated date of the MRCA of subtype 4a). This time frame precedes World War II, during which an 8000-man contingent of the Force Publique (an army composed of soldiers from the Congo serving in the Free Belgian Forces) was stationed in Egypt between 1943 and 1944 (Nigel, 1991). However, these dates can be reconciled if two or more genetically-distinct subtype 4a lineages arrived in Egypt during World War II. It is clear that the Egyptian HCV epidemic originated from more than one Central Africa strain, as subtypes 4a, 4m, 4n and 4o each independently moved to Egypt. Stronger evidence for the role of historical events in the trans-African movement of genotype 4 comes from the presence of Tunisian isolates closely related to strains from the DRC, within subtype 4k. Our molecular clock results date the MRCA of the Tunisian 4k strains to between 1928 and 1975. Tunisian troops were deployed in the DRC as part of the UN peacekeeping forces during the Congo crisis of 1960–65 (Mays, 2010) and may therefore have transported the virus to Tunisia upon their return. The movements of large numbers of troops among populations, combined with a high likelihood of blood transfusions and/or parenteral medical treatments, provided an ideal scenario for the spread of HCV out of central Africa.

Our NS5B phylogeny contains a very high diversity of genotype 4 sequences sampled from France (Supplementary. Fig. 2). This can be explained by the historical presence of the French colonial empire in central Africa, followed by more recent immigration of individuals to France from former colonies (see also Nicot et al., 2005). We also noted that subtype 4w has, to date, been found only in Portugal. Portugal was one of the first nations to have a foothold in the Congo region (Appiah and Gates, 1999) and was present in both mainland Angola and in the Cabinda exclave that lies between the DRC and the Republic of the Congo. Our phylogenetic results suggest that subtype 4w is a sub-lineage of 4b, the latter being a highly diverse subtype found in several countries, including countries that neighbour Cabinda (Fig. 3; Supplementary. Fig. 2).

Other previously unrecognised geographic trends can be discerned from our analysis. For example, the majority of isolates belonging to subtypes 4q and 4v (9 out of 14) were sampled from Rwanda and Burundi, and samples from these two countries are rarely found in other subtypes. This implies that there has been little further viral dissemination from these countries following the introduction of subtypes 4q and 4v there. The reverse pattern is observed in several adjacent countries of central Africa, specifically the DRC, Republic of the Congo, Cameroon and Gabon Multiple subtypes of genotype 4 are present in each of these countries, and sequences from them appear in all three of the intra-genotypic lineages L1, L2 and L3. This indicates that historical viral movement among these locations was comparatively common. This might be explained by historical demographic movements and trading links during the African colonial era. However, genotype 4 does not appear to have spread to the Americas via the slave trade, unlike genotype 2 which is endemic in west Africa (Markov et al., 2009). This may reflect the various ways that European colonial powers exploited different African regions; the western Atlantic coast, including the Gold Coast, was the main hub for the transatlantic slave trade, whereas Central and Eastern Africa were more commonly used to provide natural resources and labour (Suret-Canale, 1971).

Approximately 9% (74 out of 806) of all isolates examined in our study are not classified into a currently defined subtype, and all unclassified isolates were from central African countries. Detection of divergent yet uncommon strains is to be expected if HCV genotype 4 originated and has circulated endemically in central Africa for several centuries. Under this hypothesis the more prevalent subtypes of genotype 4 likely represent those few lineages that by chance were amplified by changes in human behaviour during the previous century (as previously suggested for subtypes 1a, 1b, 3a globally, and for genotype 6 in Asia; e.g. Simmonds, 2004, Magiorkinis et al., 2009, Pybus et al., 2009). We noted two lineages (4car and 4drc) that represent candidates for possible new subtypes. Full genome sequencing of isolates from 4car and 4drc would confirm their subtype status. Lineages that are currently rare and geographically-restricted could potentially spread epidemically and internationally if introduced into high risk groups, as presumably occurred to subtype 4d (De Bruijne et al., 2009, Ciccozzi et al., 2012). This risk is of particular relevance to genotype 4 which, like genotype 1, is more refractory to standard anti-viral drug treatment than other genotypes (e.g. Chen et al., 2012). There are no large scale surveys of HCV diversity in several highly populous African countries—Ethiopia, Tanzania, Angola and Chad among them—and some other countries are represented only by surveys with small sample sizes. Further screening of HCV genetic diversity in Africa is required to help plan effective treatment strategies in the region and inform future vaccine design.

## Methods

### Study population

A total of 1999 EDTA blood samples were collected from informed consenting members of the uniformed services as part of a screening programme for infectious diseases. This collection has been studied previously for HIV-1 (Djoko et al., 2011), human parvovirus 4 (Sharp et al., 2010) and human pegiviruses (Iles et al., 2013). A preliminary small scale survey (*n*=299) of this collection for HCV discovered HCV RNA in ~4% of samples (Iles et al., 2013). Collection took place during 2007 in Kinshasa, capital of the DRC. The samples were anonymised although patient year of birth were available for most. All samples were from male individuals, whose mean age was 44 (range 22–77 years).

### Screening, RT-PCR and sequencing

All samples were tested for HCV RNA. Viral RNA was extracted from sera using the Nucleospin 96 RNA kit (Macherey-Nagel) as per the manufacturer’s instructions. The reaction product was screened for HCV RNA with a one-step RT-PCR amplification of the 5′UTR region using Superscript III with Platinum *taq* (Invitrogen, Life Sciences); primers are listed in Table 1. Samples positive for HCV RNA were subsequently amplified and sequenced in the core and NS5B regions using the same enzymes as used for the 5′UTR, noted above. Controls were run in parallel at each step. Primers were obtained from previous studies or were designed using a large alignment of whole HCV genome sequences that included subtypes belonging to all 7 genotypes (Table 1). The internal primers were used for sequencing with BigDye Terminator (Applied Biosystems). Traces were examined using 4Peaks (Nucleobytes). A total of 34 core sequences (accession numbers KF813071-KF813095, KJ408429-KJ408436 and KJ416140) and 48 NS5B sequences (KF826150-KF826197) were obtained in this study.

**Table 1:** Details of primers used in this study.

**Primer name**	**Source**	**Sequence (5′-3′)**	**Position**^a^
**Murphy 5′UTR F**	Murphy et al. (2007b)	GAAAGCGTCTAGCCATGGCGTTAGT	71–95
**Murphy 5′UTR R**	Murphy et al. (2007b)	CTCGCAAGCACCCTATCAGG	311–292
**5′ UTR Ex 400F**	This study	CCTTGTGGTACTGCCTGATAG	279–299
**CHV core 980 Rex**	This study	AGTGCCARRAGGAACATAGA	883–864
**5′UTR In 405 F**	This study	CTGATAGGGTGCTTGCGAGTG	293–313
**CHV core 973 Rin**	This study	AGTGCCARRAGGAAGATAGARAA	883–861
**NS5B Ex 8274 F**	This study	TGGGGATCCCGTATGATACCCGCTGCTTTGA	8245–8275
**NS5B Ex 8616 R**	This study	CGGAATTCCTGGTCATAGCCTCCGTGAA	8643–8616
**NS5B In 8378 for**	This study	GACACCCGCTGCTTTGACTC	8259–8278
**NS5B In 8611 rev**	This study	GAGTCTTCACGGAGGCTATGACNAGGTA	8638–8611

a Numbering relative to isolate H77 (Genbank accession number AF009606).

### Sequence collation

All available genotype 4 sequences were downloaded from the Los Alamos HCV sequence database (Kuiken et al., 2005) and from GenBank. Sequences were retained if they spanned either of the two subgenomic regions sequenced in this study: core (positions 342–1265 relative to H77) or NS5B (positions 8265–8624). Only one sample per region from each infected individual was retained and sequences from non-human subjects were excluded, as were sequence fragments shorter than 200 nucleotides. We noted a disproportionate number of sequences from subtype 4a, largely resulting from the high number of published studies concerning the Egyptian HCV epidemic. To bring the number of 4a sequences approximately in line with those of other subtypes we randomly removed sequences separately from each of the three main sub-genotypic lineages of the 4a phylogeny, thereby maintaining the full genetic diversity of the subtype in our data set. We also reduced in a similar manner the disproportionate number of subtype 4d sequences sampled in France.

The reference database sequences were collated with the new sequences obtained in this study, resulting in a total of 806 isolates across all genome regions. All sequences were aligned by hand using Se-Al v2.0 (available from http://tree.bio.ed.ac.uk), resulting in a ‘core alignment’ containing 177 core sequences and an ‘NS5B alignment’ containing 765 NS5B sequences. Subsequently, a ‘concatenated alignment’ was created by combining and concatenating core and NS5B sequences if they were sampled from the same individual and covered both genome regions. The resulting joint alignment contained 136 taxa. In order to best estimate the branching order among HCV subtypes within genotype 4, we also compiled a ‘whole genome’ alignment that contained all genotype 4 reference genome sequences described in Smith et al. (2014).

For each isolate we surveyed online databases and the primary literature for two pieces of information: year of sampling and country of origin. Most isolates (83%) were sampled from African or Middle Eastern countries. A search of the primary literature revealed that some HCV strains sampled in Europe or North America represent infections from recent immigrants from Africa or the Middle East. In these instances, the ‘country of origin’ of the infection is defined as the country from which the individual emigrated. A summary of the geographic distribution of the sequences used in this study is provided in Fig. 1.

### Phylogenetic analysis

Phylogenies were estimated for the core, NS5B and whole genome alignments using maximum likelihood (ML) as implemented in GARLI v0.951 (Zwickl, 2006). The analysis used a General Time-Reversible (GTR) nucleotide substitution model, estimated base frequencies, and a gamma distribution model of among-site rate variation. Statistical support for phylogenetic clustering was calculated using an ML bootstrap approach with 500 bootstrap replicates; bootstrap scores were summarised using TreeAnnotator (http://beast.bio.ed.ac.uk/TreeAnnotator). Phylogenies were visualised and annotated using FigTree v1.4 (http://tree.bio.ed.ac.uk/software/figtree). Newly-generated sequences were classified by computing *p*-distances to the HCV subtype reference sequences provided in Smith et al. (2014). A *p*-distance threshold of <0.1 was used to assign subtypes. *p*-distances between sequences were calculated using DNAdist in the Phylip package (Felsenstein, 1989).

### Calibration of the molecular clock

Molecular clock models can be used to reconstruct the evolutionary history of HCV genotype 4 on a timescale of years, provided that estimates of the rate of molecular evolution are available for the viral genomes regions being investigated. As in previous studies, we cannot directly estimate reliable HCV evolutionary rates from the alignments in hand as the range of sampling times is too narrow (Pybus et al., 2009, Salemi and Vandamme, 2002). Therefore we estimated evolutionary rates for the core and NS5B regions using independent sets of HCV sequences that have been shown to contain good temporal information. These estimates were then used as informative prior distributions for evolutionary rate parameters in all subsequent Bayesian evolutionary analyses (see next section).

Gray et al. (2011) undertook a comprehensive analysis of HCV evolutionary rates and we use their alignments to estimate rates that are specific to the core and NS5B genome regions sequenced here (positions 342–945 and 8265–8624, respectively). We analysed two alignments, comprising 65 subtype 1a sequences and 54 subtype 1b sequences, respectively (Gray et al., 2011). These rates are likely to be accurate for our genotype 4 study because genotypes 1 and 4 are more closely related than other subtypes (Salemi and Vandamme, 2002) and because HCV evolutionary rates vary considerably more between genome regions than they do between genotypes and subtypes (Gray et al., 2011). Evolutionary rates were estimated using the Bayesian Markov Chain Monte Carlo (MCMC) inference method implemented in BEAST v1.7.5 (Drummond et al., 2012). These analyses employed a SDR06 nucleotide substitution model (two independent HKY+Γ substitution models—one for the first and second codon positions, and one for the third), an uncorrelated lognormal relaxed molecular clock model, and a Bayesian skyline plot coalescent model (Shapiro et al., 2006). Nucleotide frequencies were estimated from the data. Rates of molecular evolution were estimated for three different partitions of the whole genome alignment: (i) a core partition (sites 342–945), (ii) a NS5B partition (sites 8265–8624), and (iii) a concatenated core+NS5B partition (sites 342–945 plus 8265–8624). The rate parameters estimated for these three partitions are shown in Table 2. Each MCMC analysis was run for at least 100,000,000 states. Table 2 also includes evolutionary rate estimates for whole genome sequences, which were taken directly from Gray et al. (2011).

**Table 2:** Estimated evolutionary rate parameters.

**Genome region**	**Genome positions**^a^	**Estimated nucleotide substitution rate (subs/site/year)**	**95% credible region**
**(i) Core**	342–944	5.39×10^–4^	3.41–7.46×10^–4^
**(ii) NS5B**	8274–8612	9.87×10^–4^	6.74–14.4×10^–4^
**(iii) Concatenated (Core+NS5B)**	342–944 and 8274–8612	7.43×10^–4^	4.91–10.4×10^–4^
**(iv) Complete genome**	342–9374	13.5×10^–4^	9.97–17.0×10^–4^

a Numbering relative to isolate H77 (Genbank accession number AF009606).

**Table 3:** Subtype summary.

**Subtype**	**Samples (Core)**	**Samples (NS5B)**	**Most common sampling location of core and NS5B sequences**
**4a**	11	46	Egypt
**4b**	1	10	Democratic Republic of the Congo
**4c**	35	80	Gabon
**4d**	17	45	France
**4e**	29	142	Gabon
**4f**	16	106	Cameroon
**4g**	4	7	Gabon
**4h**	1	15	Cameroon
**4k**	23	77	Democratic Republic of the Congo
**4l**	1	10	Egypt
**4m**	2	14	Egypt
**4n**	1	7	Egypt
**4o**	3	24	Egypt
**4p**	1	13	Cameroon
**4q**	2	6	Rwanda
**4r**	12	40	Democratic Republic of the Congo
**4t**	9	31	Cameroon
**4u**	1	14	Egypt
**4v**	2	7	Rwanda
**4w**	5	3	Portugal
**4car**	0	26	Central African Republic
**4drc**	0	3	Democratic Republic of the Congo
**Unclassified**	0	45

**Table 4:** Two-letter country codes used in this study.

**Code**	**Country name**
**AR**	Argentina
**BE**	Belgium
**BH**	Bahrain
**BI**	Burundi
**CA**	Canada
**CD**	Democratic Republic of the Congo
**CF**	Central African Republic
**CG**	Republic of the Congo
**CM**	Cameroon
**CY**	Cyprus
**DE**	Germany
**DK**	Denmark
**DZ**	Algeria
**EG**	Egypt
**ES**	Spain
**FR**	France
**GA**	Gabon
**GB**	United Kingdom
**IN**	India
**IR**	Ireland
**IT**	Italy
**JP**	Japan
**MQ**	Martinique
**NL**	Netherlands
**PK**	Pakistan
**PT**	Portugal
**RU**	Russia
**RW**	Rwanda
**SA**	Saudi Arabia
**SY**	Syria
**TN**	Tunisia
**UG**	Uganda
**US**	United States of America
**XX**	Unknown
**YE**	Yemen
**ZA**	South Africa

### Bayesian evolutionary analysis

To estimate the epidemic and movement history of HCV genotype 4 we analysed the ‘whole genome’ and ‘concatenated’ alignments using the Bayesian Markov Chain Monte Carlo (MCMC) inference method implemented in BEAST v. 1.7.5 (Drummond et al., 2012).

As with the evolutionary rate estimation analyses described above, we used the SDR06 substitution model, an uncorrelated lognormal relaxed molecular clock, and a Bayesian Skyline coalescent model with 10 groups. For both the ‘whole genome’ and ‘concatenated’ data sets, Bayesian model selection tests showed that the SDR06 substitution model substantially outperformed the GTR+Γ model (Bayes Factor >100; calculated using Tracer v1.5). For both data sets, a normal prior distribution was placed on the mean evolutionary rate parameter, such that the mean and variance of the prior distribution matched the ‘concatenated’ and ‘whole genome’ rate estimates shown in Table 2. Each MCMC run contained 200 million states, sampled once every 5000 states; trees were sampled every 50,000 states. Multiple MCMC runs were calculated to ensure convergence and were combined to increase the accuracy of parameter estimates. MCMC convergence and effective sample sizes were monitored using Tracer v. 1.5. Maximum clade credibility trees were calculated and annotated using TreeAnnotator 1.7.5 (Drummond et al., 2012). FigTree v1.3.1 was used to colour lineages according to their sampling location using the parsimony criterion. We deliberately chose not to apply more sophisticated Bayesian discrete state phylogeographic models (e.g. Lemey et al., 2009) to our data. Such models are highly parametric and are unlikely to be informative when applied to phylogenies with comparatively few location state changes and no sampled sequences close to the phylogeny root (such as the tree presented in Fig. 4).

## Appendix A Supplementary materials

Supplementary Material: Supplemental Fig. 1 Estimated maximum likelihood phylogeny for the core gene alignment. Bootstrap scores are shown for each subtype cluster and for clades containing samples obtained in this study, and the phylogeny is midpoint rooted. Branch lengths are in units of expected substitutions per site (see scale bar at bottom of figure). Sequences obtained in this study are marked with a red circle; those obtained from the same population during a pilot study (Iles et al., 2013) are marked with a blue circle. Sequences are labelled as follows: subtype, sampling location using two-letter country codes (ISO 3166), sampling date, isolate name, accession number. XX represents an unknown location. Subtypes are indicated with grey bars on the right side of the diagram. Subtypes in parentheses denote isolates whose core and NS5B subtypes are discordant. The subtype in parenthesis represents the subtype of the corresponding NS5B sequence. The four clusters of samples obtained in this study discussed in the main text are labelled C1, C2, C3, and C4.

Supplementary Material: Supplemental Fig. 2 Estimated maximum likelihood phylogeny for the NS5B gene alignment. Subtypes in parentheses denote isolates whose core and NS5B subtypes are discordant. The subtype in parenthesis represents the subtype of the corresponding core sequence. See the legend of Supplemental Fig. 1 for further figure details.

Supplementary Material: Supplemental Fig. 3 Estimated maximum likelihood phylogeny for the whole genome alignment. Bootstrap scores >70% are shown next to each node and the phylogeny is rooted using a genotype 1 outgroup. Branch lengths are in units of expected substitutions per site (see scale bar at bottom of figure). Sequences are labelled with their subtype and accession number. Subtypes are indicated on the right side of the diagram. The three intra-genotypic lineages discussed in the main text are labelled L1, L2, and L3.

## Footnotes

Appendix A Supplementary data associated with this article can be found in the online version at doi:10.1016/j.virol.2014.07.006.
