# Supplementary figures and images for: Phylogeography and epidemic history of hepatitis C virus genotype 4 in Africa
Source: Virology. 2014 Sep;464-465:233–43. doi: 10.1016/j.virol.2014.07.006 (PMC4162651; doi:10.1016/j.virol.2014.07.006)

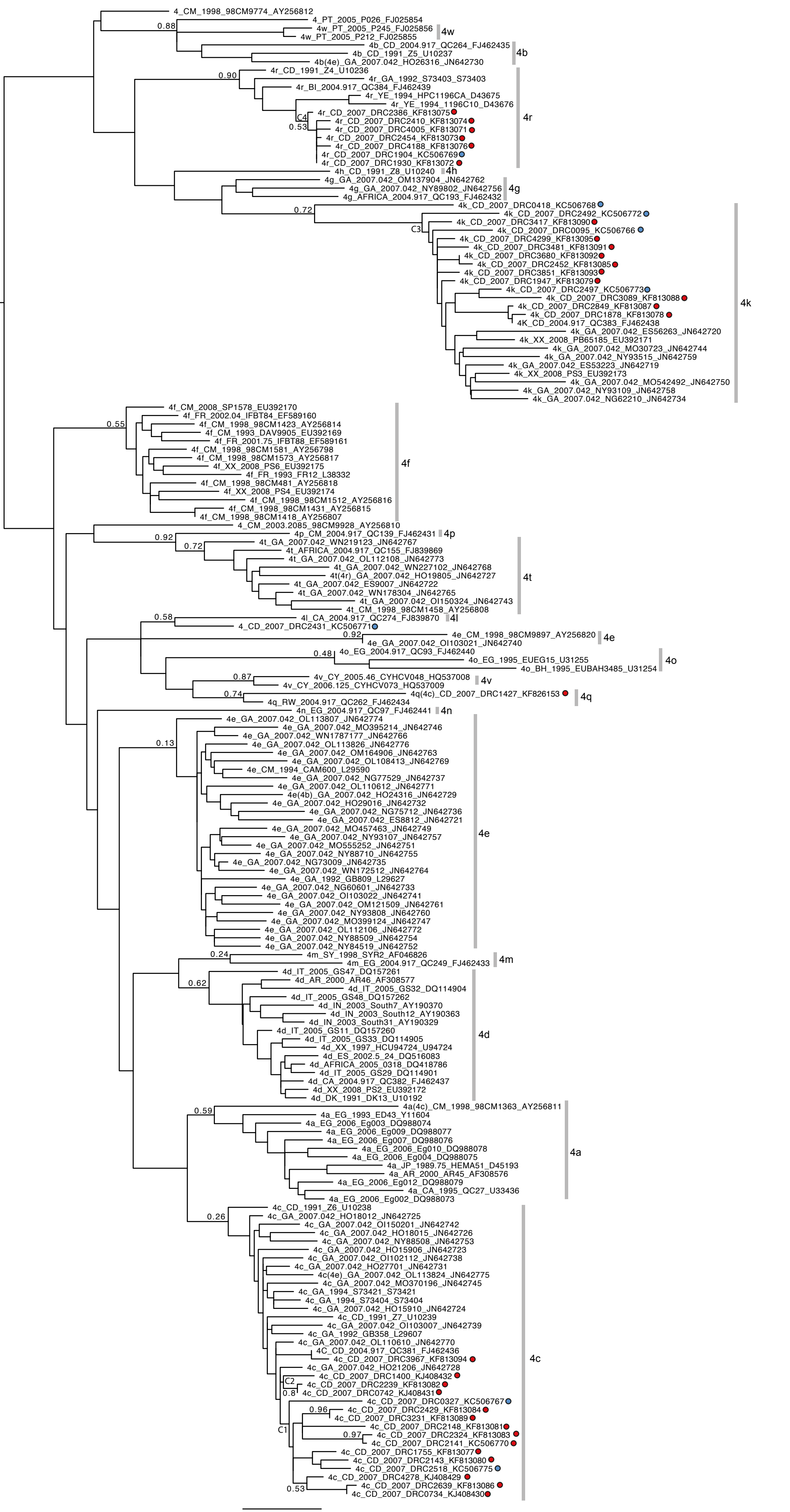

0.04

Supplement: Supplementary file 1 — Supplementary Material: Supplemental Fig. 1 Estimated maximum likelihood phylogeny for the core gene alignment. Bootstrap scores are shown for each subtype cluster and for clades containing samples obtained in this study, and the phylogeny is midpoint rooted. Branch lengths are in units of expected substitutions per site (see scale bar at bottom of figure). Sequences obtained in this study are marked with a red circle; those obtained from the same population during a pilot study (Iles et al., 2013) are marked with a blue circle. Sequences are labelled as follows: subtype, sampling location using two-letter country codes (ISO 3166), sampling date, isolate name, accession number. XX represents an unknown location. Subtypes are indicated with grey bars on the right side of the diagram. Subtypes in parentheses denote isolates whose core and NS5B subtypes are discordant. The subtype in parenthesis represents the subtype of the corresponding NS5B sequence. The four clusters of samples obtained in this study discussed in the main text are labelled C1, C2, C3, and C4. [file mmc1.pdf]

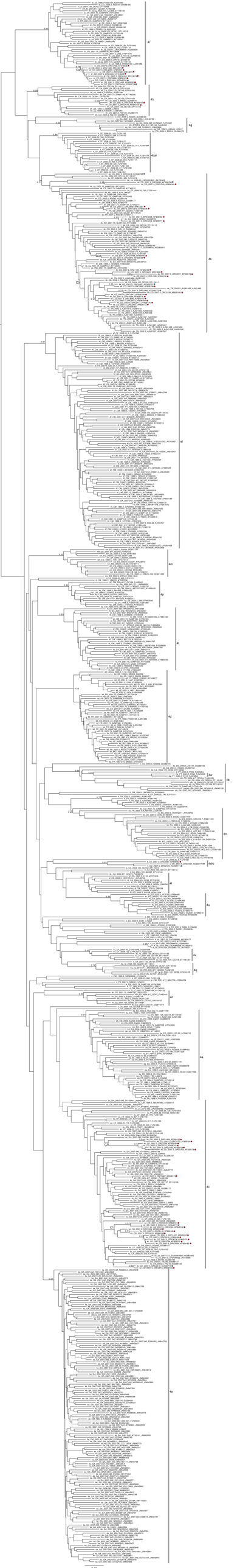

Supplement: Supplementary file 2 — Supplementary Material: Supplemental Fig. 2 Estimated maximum likelihood phylogeny for the NS5B gene alignment. Subtypes in parentheses denote isolates whose core and NS5B subtypes are discordant. The subtype in parenthesis represents the subtype of the corresponding core sequence. See the legend of Supplemental Fig. 1 for further figure details. [file mmc2.pdf]

Genotype 1

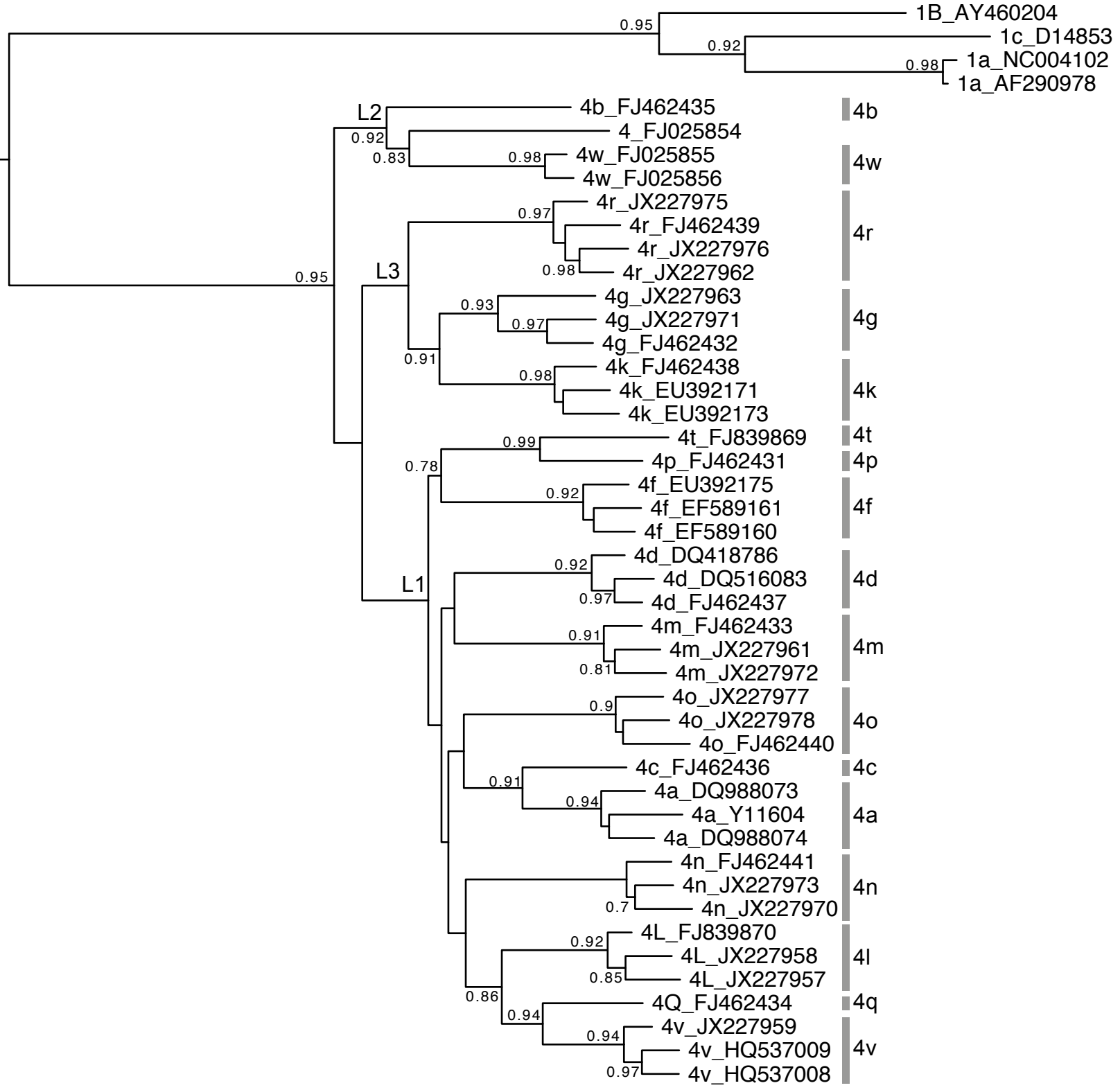

0.1

Supplement: Supplementary file 3 — Supplementary Material: Supplemental Fig. 3 Estimated maximum likelihood phylogeny for the whole genome alignment. Bootstrap scores >70% are shown next to each node and the phylogeny is rooted using a genotype 1 outgroup. Branch lengths are in units of expected substitutions per site (see scale bar at bottom of figure). Sequences are labelled with their subtype and accession number. Subtypes are indicated on the right side of the diagram. The three intra-genotypic lineages discussed in the main text are labelled L1, L2, and L3. [file mmc3.pdf]
